# Increased Cortisol Response and Low Quality of Life in Women Exposed to Intimate Partner Violence With Severe Anxiety and Depression

**DOI:** 10.3389/fpsyt.2022.898017

**Published:** 2022-06-27

**Authors:** Beatriz Cerda-De la O, Ana Lilia Cerda-Molina, Lilian Mayagoitia-Novales, Margarita de la Cruz-López, Marcela Biagini-Alarcón, Erika Lucia Hernández-Zúñiga, Javier I. Borráz-León, Jesús Alfredo Whaley-Sánchez

**Affiliations:** ^1^Clínica de Género y Sexualidad, Dirección de Servicios Clínicos, Instituto Nacional de Psiquiatría Ramón de la Fuente Muñiz, Ciudad de México, México; ^2^Departamento de Etología, Dirección de Investigaciones en Neurociencias, Instituto Nacional de Psiquiatría Ramón de la Fuente Muñiz, Ciudad de México, México

**Keywords:** intimate partner violence, cortisol response, anxiety, depression, quality of life, suicide thoughts

## Abstract

**Background:**

Intimate partner violence (IPV) is one of the most prevalent forms of violence against women globally and it is considered a public health problem. Because the experience of IPV is stressful and traumatic for victims, they are at high risk of developing alteration of the Hypothalamus–Pituitary–Adrenal (HPA) axis functioning as well as anxiety and depression symptoms. The aim of this study was to compare the quality of life and changes in cortisol response to an acute stressor between women exposed to IPV and non-exposed women. Differences according to symptoms of anxiety and depression including the risk of suicide thoughts, were also analyzed.

**Method:**

Our sample size consisted of 130 women (ages 18–68) grouped as follows: 71 women experiencing IPV and 59 women without history of IPV as control group. All participants completed a battery of questionnaires including IPV exposure, anxiety, and depression symptoms (Beck Inventories), as well as quality of life (WHOQOL-BREF). Salivary cortisol levels in response to a cognitive test with verbal, mathematical, and abstract reasoning were measured at four time points.

**Results:**

Women exposed to IPV, with severe anxiety and depression symptoms as well as suicide thoughts, exhibited heightened cortisol response after the cognitive test and reported lower quality of life compared to (i) women experiencing IPV with moderate symptoms of anxiety and depression, who showed a blunted response, and (ii) women without history of IPV with minimal to moderate symptoms, who showed a decreased cortisol profile. Social relationships dimension was in particular the most affected aspect of quality of life.

**Conclusions:**

Our findings highlight the role of cortisol responses as a complementary biological marker to be associated with severe psychiatric disturbances in women exposed to IPV.

## Introduction

Intimate partner violence (IPV) is one of the most prevalent forms of violence against women worldwide, since around one third (27%) of women aged 15–49 years has been exposed to any kind of violence at least once in their life (1). IPV is defined by the World Health Organization (WHO) (2) as “any behavior within an intimate relationship that causes physical, sexual, or psychological harm.” It includes acts of physical aggression (slapping, hitting, kicking and beating), sexual coercion (forced intercourse), emotional or psychological abuse (insults, constant humiliation, intimidation, threats of harm, or to take away children), and/or controlling behaviors (isolation from family and friends, monitoring their movements, restricting access to financial resources, employment, education or medical care) (1), which are commonly perpetrated by current or former romantic partners, even without cohabitation (3). IPV constitutes a multifactorial global public health problem affecting the quality of life of victims and their families, regardless of the country, socioeconomic status, ethnicity, or religion (4, 5). In the United States, the lifetime prevalence of IPV experienced by women is 37.3% (6) whereas the percentage is almost double in Mexico. The latest national survey in Mexico reported that 66.1% (30.7 million out of 46.5 million women) have faced violence of any kind and from any aggressor at some time in their life, whereas 43.9% have faced aggression from their current or former husband or romantic partner throughout their relationship (7). IPV affects the sense of wellbeing and causes adverse physical and mental health outcomes for victims and their families, even after the violence has ended (8, 9). For example, extensive literature has showed that being a victim of IPV is a strong risk factor for developing stress-related diseases and disorders such as gastrointestinal complains, major depressive disorder (MDD), anxiety, post-traumatic stress disorder (PTSD), substance abuse problems, and higher recurrence of suicide thoughts than women who do not suffer violence (3, 10–14). Thus, it is not surprising that the health consequences associated with IPV are among of the most common reasons for requesting medical services (8).

Stressful and traumatic life events, such as IPV are associated with alterations in the regulation of the hypothalamic-pituitary-adrenal (HPA) axis, and consequently in cortisol secretion, the end product of the HPA axis (15). For example, previous studies have evidenced either abnormally high cortisol secretion (hyper-reactivity) or reduced secretion (hypo-reactivity) in response to long-term stressors. Hyperactivation of the HPA axis has been associated with MDD (16–19) and is mainly due to an impaired negative brain feedback mechanism necessary for the recovery of basal cortisol levels after the exposure to a stressor. This altered mechanism of cortisol regulation could lead to deleterious effects on mood and cognitive performance (20). In contrast, other mental health disorders, such as PTSD, are associated with decreased responsiveness to stress and consequently with hypo-secretion of cortisol (21). Besides its function in stress regulation, cortisol has an important metabolic role necessary for the maintenance of body homeostasis (15), due to the well-characterized diurnal rhythm secretion with a normal peak around 30 min after awakening (22). Previous research on victims of IPV has focused on studying cortisol awakening response and diurnal changes, with mixed results depending on the associated mental health disorder. For instance, some studies have found lower levels of morning cortisol in women victims of IPV with psychiatric disorders such as PTSD (23), dissociative symptoms (24), or MDD (25) [but see (26) for opposite results]. Other studies have found higher levels of evening cortisol in women victims of IPV with abuse antecedents, relative to non-abused women (27), and in women with PTSD than in women without this disorder (28). Similarly, Johnson et al. (29) observed that women with PTSD had higher awakening cortisol response than women without PTSD, whereas reported chronic abuse was associated with lower levels of this hormone. To date, the only study investigating cortisol responses to a psychosocial stressor in women exposed to interpersonal violence/trauma, found higher cortisol response to the first of four tests that make up the Trier Social Stress Tests (TSST) among trauma exposed women with current PTSD than among women without PTSD (with no cortisol response) and non-traumatized controls, who showed a blunted response (30). Therefore, to contribute with the understanding of the consequences of altered HPA axis responses in women victims of IPV, we aimed to assess quality of life as well as cortisol levels in response to a cognitive test in a group of female psychiatric patients exposed to IPV with anxiety and depression symptoms, and to compare them with a group of women without history of IPV. In addition, we sought to evaluate potential differences in the results according to recurrency of suicidal thoughts. According to the literature, we expected that women experiencing IPV have a higher cortisol response, worse quality of life, and more severe symptoms of anxiety and depression than women without IPV. We also expected a much higher cortisol response in those patients with suicide thoughts.

## Methods

### Participants

This was a cross-sectional study that included a total of 130 adult women with an age range of 18–68 years (Table 1) as follows: 74 female psychiatric patients, diagnosed with anxiety and depression, who met the inclusion criteria of being currently receptors of IPV, were recruited during the first admission at the Gender and Sexuality Clinic of the Instituto Nacional de Psiquiatría “Ramón de la Fuente Muñiz,” in Mexico City, Mexico. Three women were excluded from the sample because they did not complete the entire psychological instruments; thus, the final sample in this group was 71 women (hereinafter IPV women). As controls, female students, and administrative employees (non-involved in the clinical research and who have no contact with psychiatric patients) of the same Institute were invited to participate. A total of 59 women who met the inclusion criteria of not being exposed to IPV were included in the control group. Demographic characteristics of both groups (age, education, occupation, and marital status) can be found in Table 1. Exclusion criteria in both groups were comorbidity with psychotic disorders, intellectual disability, substance use, serious physical illness, and those conditions affecting cortisol levels such as pregnancy, using birth control pills, and current treatment with anti-inflammatories. Diagnoses were made by the Psychiatrists and Psychologists in charge of the Gender and Sexuality Clinic according to the Diagnostic and Statistical Manual Version 5 (DSM-5) (31). Before signing the informed consent letter, all participants received detailed information about the protocol and purposes of the study and were told that they had the right to finish the procedure at any time without any negative consequence for them. This study took place from 2018 to the beginning of 2020, prior to the COVID-19 pandemic which discards the effects of the pandemic on our results.

**Table 1:** Demographic data and descriptive statists of the participants (IPV vs. control non-IPV).

	**IPV (*N* = 71)** **frequency (%)**	**Non-IPV (*N* = 59)** **frequency (%)**	***X*** ^**2**^	**Sig**.
Age	44.37 (10.85)	32.08 (14.46)	30.28	<0.001
Alcohol	13 (18.3%)	38 (64.4%)	29.76	<0.001
Smoke	11 (15.5%)	18 (30.5%)	4.42	0.035
**Marital status**			37.5	<0.001
Single/relationship	9 (12.7%)	36 (61.0%)		
Married/cohabiting	45 (63.4%)	22 (37.3%)		
Divorced/separated	17 (23.9%)	1 (1.7%)		
**Occupation**			48.77	<0.001
Housewife	40 (56.3%)	2 (3.4%)		
Formal employment	10 (14.1%)	8 (13.6%)		
Informal^a^ employment	12 (16.9%)	15 (25.4%)		
Student	9 (12.7%)	34 (57.6%)		
**Education degree**			28.50	<0.001
Elementary	10 (14.1%)	0		
Secondary	22 (31.0%)	6 (10.2%)		
High School	21 (29.6%)	13 (22.0%)		
University/master	18 (25.4%)	40 (67.8%)		
**Types of violence**				
Psychol	6 (8.45%)	–		
Sexual	1 (1.40%)	–		
Psychol & sexual	3 (4.22%)	–		
Psychol & physical	5 (7.04%)	–		
Psychol & physical & physical severe^b^	4 (5.63%)	–		
Psychol & sexual & physical	19 (26.76%)	–		
The four types	33 (46.47%)	–		

*Data express frequency and percentage, excepting for age expressed as mean and standard deviation of the mean (SD)*.

a *Informal Employment Refers to a job Without Social Security*.

b *The Type of Physical Violence Where Life Has Been at Risk. (Psychol = Psychological)*.

### Ethics

This study was approved by the Research and Ethic Committees (Project Number: SC19114.0) and conducted in compliance with the declaration of Helsinki and the National Official Norms for Research with Human Beings (NOM-012-SSA3-2012). As compensation, patients were offered to receive free psychotherapeutic interventions; control women were offered to receive their psychological and endocrine profiles and were also offered to receive free psychological assistance if needed.

### Assessment of Intimate Partner Violence

Exposition to intimate partner violence was assessed using the Mexican Scale of Violence by the Male Partner Against Women (32). This is a 27-item scale (Cronbach's α = 0.99) to measure the types of violence currently experienced and their severity within the last 12 months (psychological, sexual, physical, and severe physical violence –where life has been at risk–). The severity index is calculated by summing the scores of the frequency of each event (i.e., never = 0, sometimes = 4–9, many times = 8–14, several times = 12–27). According to the weight assigned to each item, the index is classified as: no violence, moderated violence, and severe violence (Cronbach's α for the present sample = 0.89). Table 1 indicates the frequency and percentages of the types of violence reported by IPV women. Only 7 out of 71 reported one type of violence, the other patients reported two, three, and even the four types of violence.

### Assessment of Anxiety and Depression

We used the self-reporting Spanish version of the Beck Depression Inventory (BDI) (33) and Beck Anxiety Inventory (BAI) (34). The total score of both inventories ranges from 0 to 63. The validated version of BDI (Cronbach's α = 0.87) establishes 0–9 points as minimal o absent depression, 10–16 mild, 17–29 moderate, and >29 severe. Cronbach's α in the present study was 0.88. The validated version of BAI (Cronbach's α = 0.83) considers 0–5 points as minimal o absent anxiety, 6–15 mild, 16–30 moderate, and >30 severe. Cronbach's α in the present study was 0.93.

Most of the IPV patients (95.77%, *N* = 68) had symptoms of both anxiety and depression, and 29 patients (40.8%) answered the item 9 of the BDI scale about suicidal thoughts (see Table 2). These 29 women were at the highest limit of moderate depression (mean score 28.76 ± 9.48) but had a mean score of severe anxiety (32.03 ± 11.12). Patients without suicide thoughts had moderate symptoms (depression: 18.67 ± 6.94; anxiety: 23.60 ± 13.17). Most of the women without IPV (94.9% *N* = 56) had minimal/absent to moderate symptoms of both anxiety and depression (Table 2).

**Table 2:** Descriptive statistics for anxiety, depression, and quality of life applied to the participants (IPV vs. control non-IPV).

	**IPV (*N* = 71)** **Mean ±SD (*N*)**	**Non-IPV (*N* = 59)** **Mean ±SD (*N*)**	***F***	**Sig.**
**Anxiety (BAI)**	27.04 ± 12.98	11.71 ± 9.27	50.76	<0.001
Minimal/absent	4.40 ± 0.54 (5)	2.05 ± 1.87 (19)		
Mild	10.78 ± 2.9 (9)	10.0 ± 2.96 (22)		
Moderate	22.64 ± 4.59 (25)	23.78 ± 3.99 (18)		
Severe	38.59 ± 7.02 (32)	0		
Suicide thoughts	32.03 ± 11.12 (29)	0		
**Depression (BDI)**	22.79 ± 9.44	10.09 ± 6.87	74.76	<0.001
Minimal/absent	5.20 ± 2.28 (5)	4.47 ± 3.09 (32)		
Mild	12.13 ± 2.35 (8)	12.69 ± 1.66 (16)		
Moderate	21.93 ± 3.33 (42)	20.82 ± 3.54 (11)		
Severe	35.88 ± 5.77 (16)	0		
Suicide thoughts	28.76 ± 9.48 (29)	0		
General QL	2.35 ± 0.86	3.50 ± 0.92	53.05	<0.001
Health QL	2.26 ± 0.98	3.31 ± 0.90	38.55	<0.001
QL Physic	2.71 ± 0.67	3.71 ± 0.74	64.42	<0.001
QL Psychological	2.42 ± 0.67	3.52 ± 0.74	78.64	<0.001
QL Environment	2.55 ±0.55	3.41 ± 0.85	48.08	<0.001
QL Social	1.51 ± 0.56	3.58 ± 1.08	195.33	<0.001

*BAI, Beck anxiety inventory; BDI, Beck depression inventory; QL, Quality of life WHOQOL-BREF inventory (mean of the Likert scale)*.

### Assessment of Quality of Life

Quality of life was evaluated using the 26-item Quality of Life questionnaire (WHOQOL-BREF) (35, 36). This scale measures four health dimensions: Physical (e.g., activities of daily living, energy, and fatigue); Psychological (e.g., bodily image and appearance, positive and negative feelings); Social relations (personal relationships, social support, and sexual activity); Environment (e.g., financial resources, home environment, transport), and two general questions: (1) How do you rate your quality of life in general? (2) How satisfied are you with your health? Each item was rated on a 5-point Likert-type scale (1 = very bad/completely unsatisfied; 3 = neither unsatisfied/neither satisfied; 5 = very good/completely satisfied). This scale has an internal consistency range of 0.68–0.81 being the lowest for social relations (35). In the present sample, the internal consistencies were: total scale α = 0.88; physical health α = 0.72; psychological health α = 0.73; environment α = 0.70; social relations α = 0.60.

### Cortisol Response

Saliva samples were subjected to two subsequent freeze-thaw cycles to free them from mucopolysaccharides and proteins (37). After each thawing, samples were centrifuged at 1,500 g during 30 min at 4°C; the supernatants were collected and frozen again. We measured cortisol in duplicates using commercially available kits (ENZO Life Sciences) through the ELISA technique by following the manufacturer's instructions. Cortisol concentrations were reported in pg/ml. Inter-assay and intra-assay coefficients were 9.8 and 7.1%, respectively.

### Procedure

Participants were previously instructed to brush their teeth before coming to the test and not to eat, smoke, drink tea, coffee, or any sweet or colored beverage (only water), for at least 2 h before the test. First, participants completed the general information questionnaire; after that they collected a first saliva sample (2-3 ml) by passive drool into a new polypropylene tube (basal sample). After the first saliva sample was collected, participants were exposed to an acute stressor as follows: they were instructed to complete a brief IQ test comprising questions about verbal, mathematical, and abstract reasoning. Participants were told that they had exactly 10 min to complete the test and that two psychiatrists would be taking care not to cheat on the test. 15, 30, and 45 min after the test, the second, third, and fourth saliva samples were collected, and volunteers completed the inventories described above. All the procedure took about 60–70 min and was performed in groups of 10–12 participants per session. Saliva samples were labeled with a code to ensure the confidentiality of the volunteer and immediately frozen and stored at −20°C until assayed.

### Statistical Design

Differences between groups (i.e., IPV women vs. non-IPV women) in demographic data, psychiatric scales and quality of life were analyzed with *X*^2^ and one-way ANOVA. To analyze the cortisol response, we used a Generalized Equation Model (GEE) suitable for data dependency, i.e., repeated samples design (38). Cortisol levels (log transformed to normalize the distribution) were introduced as dependent variable; as independent factors we included group (IPV vs. non-IPV) and time of saliva sample (basal, 15, 30, and 45 min *post-test*), whereas age (in years) was included as covariate. Because anxiety and depression scores were highly correlated (Pearson *r* = 0.798, *N* = 130, *p* < 0.001) and most of the participants had both symptoms, we summed anxiety and depression scores of each participant, to create only one continue variable reflecting the severity of the symptoms; this variable was also included as covariate in the model. We analyzed the main effects and interactions. To compare the quality of life between IPV women with and without suicide thoughts, we used a Multivariate GLM (MGLM) analysis suitable for multiple dependent variables. The data were analyzed using SPSS version 22 (SPSS Inc., Chicago, IL, USA). We used Bonferroni as *post-hoc* test and significance was set at *p* ≤ 0.05. Results are expressed as the mean ± SD, and all the graphics express mean and 95% CI.

## Results

### Demographics and Quality of Life (IPV vs. Non-IPV Women)

Statistically significant differences between women with and without IPV were found (Table 1). In general, IPV patients were older (*M* = 44.37 vs. 32.08 years), consumed less alcohol and tobacco than control women. Most of the IPV patients were married/cohabiting, housewives, and had middle school education. Table 2 shows that IPV patients had significantly more symptoms of anxiety and depression, ranging from moderate to severe levels, whereas most women without IPV ranged from minimal to moderate levels (none of the control women had severe symptoms or suicide thoughts). Regarding quality of life, women with IPV reported a poorer quality of life in all the domains than control women; this was especially pronounced for the “social relations” domain, reporting a mean score of 1.5, corresponding to a completely unsatisfied level (Table 2).

### Cortisol Response (IPV vs. Non-IPV Women)

**Figure 1:**
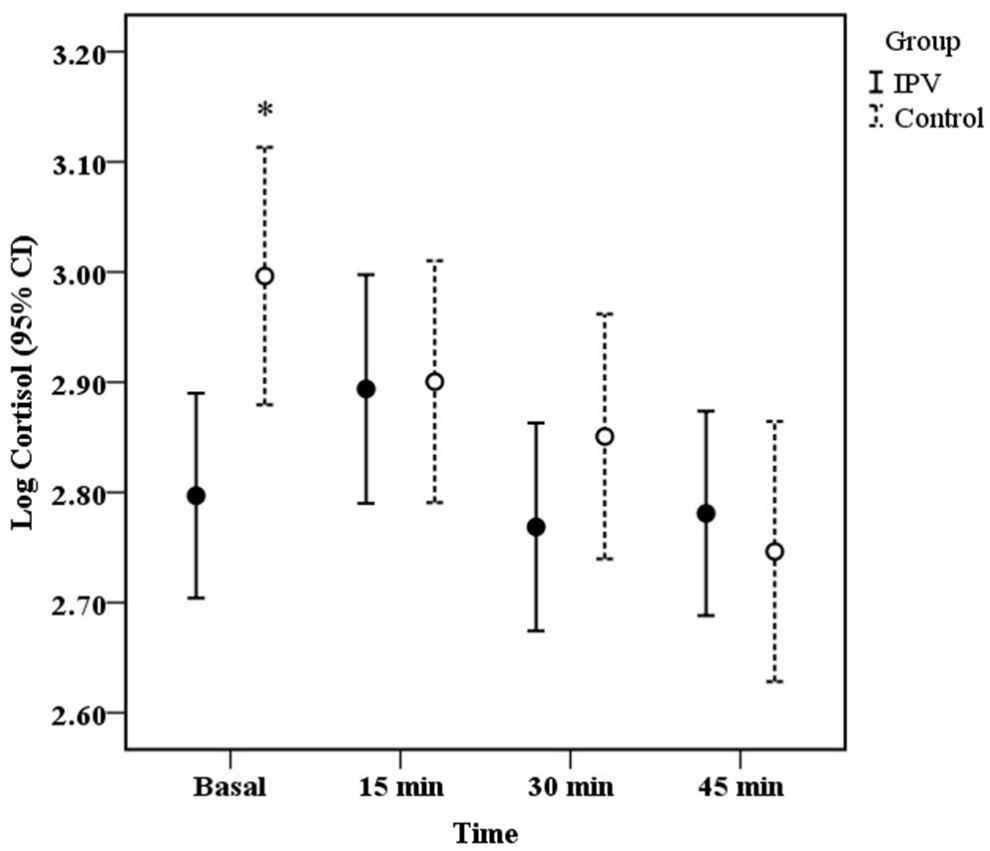
Mean (± 95% CI) of salivary cortisol concentration before (basal), and 15, 30, 45 min after the onset of the cognitive test in women with IPV (bold circle, *N* = 71) and control without IPV (open circle, *N* = 59). *P < 0.05 vs. 45 min.

The GEE Model showed a significant effect of time (*X*^2^ Wald = 14.71, d.f. = 3, 129, *p* = 0.002), whereas the group alone did not (*X*^2^ Wald = 0.005, d.f, = 1, 129, *p* = 0.92); however, the interaction time × group was statistically significant (*X*^2^ Wald = 11.23, d.f. = 3, 129, *p* = 0.01), indicating differences in cortisol response between IPV and non-IPV women. No significant effects of age (*X*^2^ Wald = 0.18, d.f. = 1, 129, *p* = 0.67) or symptomatology of anxiety and depression (*X*^2^ Wald = 2.31, d.f. = 1, 129, *p* = 0.12) on cortisol response were found. Control non-IPV women displayed a pattern of cortisol hyporeactivity whereas IPV women showed a slight non-significant increase in cortisol levels. Figure 1 indicates a statistically significant decrease in cortisol levels 45 min after the cognitive test compared to basal cortisol level in control non-IPV women (*p* = 0.01). No significant changes in cortisol levels were found for IPV women.

### Cortisol Response in IPV Women With and Without Suicide Thoughts

**Figure 2:**
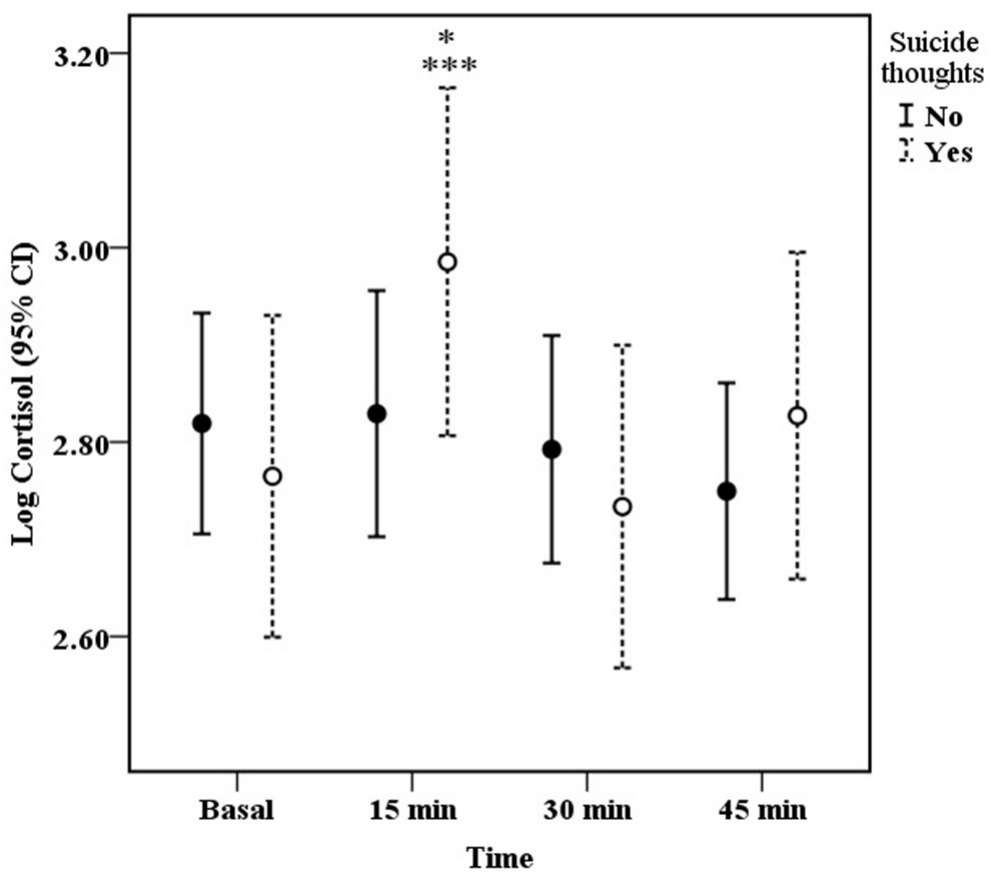
Mean (± 95% CI) of salivary cortisol concentration before (basal), and 15, 30, 45 min after the onset of the cognitive test in IPV exposed women with (*N* = 29) and without (*N* = 42) suicide thoughts. Suicide thoughts: ***P < 0.001 vs. 30 min, *P < 0.05 vs. basal.

The GEE model indicated that the interaction time × suicide thoughts was statistically significant (*X*^2^ Wald = 11.32, d.f. = 3, 71, *p* = 0.01). Figure 2 shows that women with suicide thoughts had a significant increase in cortisol levels 15 min after the cognitive test compared to basal and 30 min levels (*p* = 0.02 and *p* < 0.001, respectively). No significant differences in cortisol secretion were found for IPV patients without suicide thoughts.

### Quality of Life in IPV Women With and Without Suicide Thoughts

**Figure 3:**
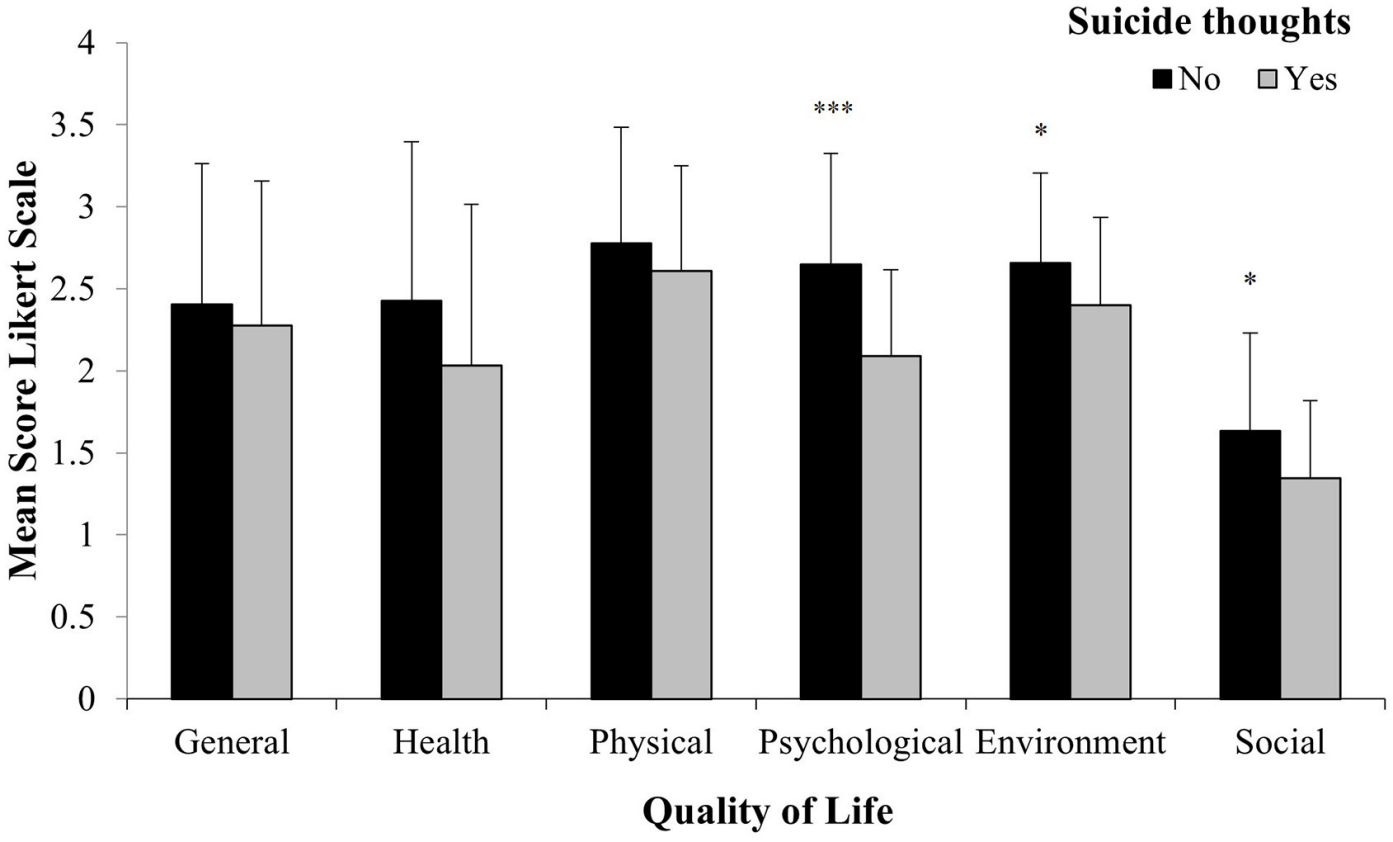
Mean (± SD) of quality of life level in IPV exposed women with (*N* = 29) and without (*N* = 42) suicide thoughts. ***P < 0.001, *P < 0.05 vs. suicide thoughts.

The results of the MGLM indicated that IPV women with suicide thoughts had significantly worse quality of life than IPV women without these thoughts (Pillai's Trace, *F* = 3.15, d.f. = 6, 64, *p* = 0.009, η = 0.22, power = 0.89). Inter-subject analyses indicated that the dimensions of psychological, environment, and social quality of life were significantly lower in IPV women with suicide thoughts (*F* = 14.05, *p* < 0.001; *F* = 3.85, *p* = 0.05; *F* = 4.69, *p* = 0.03, respectively, see Figure 3).

## Discussion

The present study aimed to compare cortisol responses between women exposed to IPV and women without IPV, with women in both groups showing symptoms of anxiety and depression. Our findings indicated that cortisol responses in IPV women depended on the exhibition of suicide thoughts and the severity of anxiety and depression symptoms. IPV women with suicide thoughts and severe anxiety and depression symptoms had an increased cortisol response 15 min after the cognitive test compared to IPV women without suicide thoughts and mild to moderate symptoms. Contrastingly, control women without IPV who exhibited minimal to moderate symptoms, showed a decreased cortisol profile. These results support the notion that the experience of IPV is highly stressful for victims (3, 10, 12). Our findings also showed a worse perception in the quality of life in IPV women compared to non-IPV ones, especially for social relationships, which may reflect potential negative effects of their social environment such as experiencing low social support. This result is consistent with previous research asserting that one strategy of batterers to exert control over their partner is to isolate them from family and /or friends, as well as to impose financial restrictions (39, 40) which can diminish their support perceptions.

Our results about cortisol responses in IPV women with suicide thoughts and severe anxiety and depression, agree with previous literature reporting that depressed patients tend to exhibit a hyperactivation of the HPA axis with increased cortisol levels, probably as a result of their psychological load (10, 41). The role of depression and anxiety as comorbidities has also been reported. For instance, Young et al. (42) found an increased activity in the HPA axis of depressed people with comorbid anxiety disorders compared to control subjects.

Our findings are also in line with those reported by Morris et al. (30) who found cortisol responses to the first stage of the TSST to be more pronounced in trauma survivors with current PTSD compared to those without PTSD and non-traumatized controls (who showed blunted responses). It has been reported that people with MDD may have impaired stress coping strategies, partly as a consequence of an increased sensitivity of the HPA axis activation along with an impairment of the negative feedback regulation responsible for ending the stress response (41, 43). In contrast, IPV women with moderate symptomatology and without suicidal thoughts had a blunted response, possibly indicating a higher sensitivity for a negative feedback regulation of cortisol which could contribute to explain the relatively better quality of life reported (44). Previous literature has informed that both exaggerated and blunted cortisol responses after acute stress exposure has been associated with adverse health outcomes (45); in the present study, IPV women indicated poor self-perceptions of general health and quality of life. These perceptions were even more pronounced in the case of IPV women with suicide thoughts and higher cortisol reactivity. This finding agrees with research associating a high degree of chronic stress with recurrent depressive episodes (46). Contrastingly, some clinical studies have associated a blunted cortisol response to stress with poor cognitive abilities, addiction, and poor self-reported health (47). Although some literature has previously discussed the implications for overall wellbeing and health of experiencing either hypo or hyper-reactivity of cortisol levels in response to acute stressors, our results indicate that higher cortisol responses (i.e., hyper-reactivity), may have more negative outcomes including the risk of suicide thoughts and higher symptoms of anxiety and depression (45). The fact that non-IPV women with minimal to moderate symptoms of anxiety and depression had a decreased cortisol secretion supports the hypothesis of an adaptive mechanism promoting rapid negative feedback regulation, which protects the brain from the negative effects of chronic high levels of cortisol (48–50). For instance, Elzinga et al., (51) has suggested that reduced cortisol response in non-clinical women could be explained by an enhanced stress resistance or a marker for resilience. Furthermore, it has been suggested that cortisol hyper-reactivity characterize the short-term effect of an acute stress at the beginning of the adversity, whereas hypo-reactivity may develop in the long-term (48). According to the allostatic load model, individuals who show greater difficulty in adapting to repeated stressors may fail to develop a proper habituation, leading to a higher allostatic load which contribute to hyper-reactivity of the HPA axis and the consequent negative effects on psychological wellbeing and general health (52, 53). In this regard, some studies have shown that most healthy subjects exhibit HPA habituation across repeated psychosocial stressors; however, individual differences may explain variations in sensitization levels (54). Contrastingly, other studies have found that diminished cortisol secretion in response to early trauma exposure increases the risk for developing some mental disorders such as PTSD (55, 56). Discrepancies in the results might be related in part to the nature of the laboratory stressor and the psychological condition of the study subjects. It has been documented that social-evaluative laboratory tests such as the TSST, are perceived as threats by interpersonal trauma survivors with some mental disorder, showing exaggerated responses compared to healthy controls. Thus, people with a history of repeated stressors or negative life events but without developing any mental disorder, might exhibit lower HPA reactivity (51, 57). This hypothesis may explain our findings for the control non-IPV women. It is important to note that our studied population had different sociodemographic characteristics, for example, whereas most IPV women were housewives, non-IPV were mainly students, and also IPV women had less education level than non-IPV. The fact that non-IPV women had symptoms of depression and anxiety could be explained by the fact that most of them were students and could be exposed to academic stress; however, none of these women presented neither severe symptoms nor suicide thoughts. The diminished cortisol response in non-IPV women supports our explanation.

Overall, our results support previous evidence on the negative impact of IPV on women's mental health and quality of life. Particularly, we found that IPV women have markedly higher score of anxiety and depression than control women without IPV, and a poor perception of the quality of life that worsens with the exhibition of suicidal thoughts in patients with severe symptoms. The high cortisol response in those patients, might signal a reduced ability to contend with acute stressors of daily life challenges with potential long-term negative effects in their lives. The findings presented in this study are highly relevant for psychiatrist and clinicians because they highlight the need to design interdisciplinary therapeutic strategies that contemplate the inclusion of physiological variables (e.g., cortisol responses) as complementary markers of general and mental health of victims of IPV. Since there is evidence of the beneficial impact on general health resulting from the availability of social support, further research is needed to test the long-term effects of therapeutic strategies aimed to restore or increase social support in IPV women on mental health and cortisol responses.

## Limitations

The small sample size both in IPV patients and control women without IPV as well as the age differences between both groups may have represented a limitation in the present study. Even though we did not find a significant effect of age in the results, the hypotheses examined in this study have to be tested in future research where this limitation is taken into account. Moreover, the inclusion of sex hormones such as estradiol has to be addressed in further research since it has been shown that fluctuations in this hormone might alter the HPA axis in women with menopausal symptoms (58).

## Data Availability Statement

The raw data supporting the conclusions of this article will be made available by the authors, without undue reservation.

## Ethics Statement

The studies involving human participants were reviewed and approved by Comité de Ética en Investigación. The patients/participants provided their written informed consent to participate in this study.

## Author Contributions

BC-D, AC-M, MC-L, MB-A, and JW-S designed the proposal, recruited the participants, contributed to the analyses, and writing and interpretation of the data. LM-N measured hormones and contributed to the data analyses. EH-Z contributed to the application of the scales. JB-L contributed to the writing—original draft preparation. All authors contributed to the article and approved the submitted version.

## Funding

This research did not receive any specific grant from funding agencies in the public, commercial, and not-for-profit sectors; it was completely financed by the Instituto Nacional de Psiquiatría Ramón de la Fuente Muñiz.

## Conflict of Interest

The authors declare that the research was conducted in the absence of any commercial or financial relationships that could be construed as a potential conflict of interest.

## Publisher's Note

All claims expressed in this article are solely those of the authors and do not necessarily represent those of their affiliated organizations, or those of the publisher, the editors and the reviewers. Any product that may be evaluated in this article, or claim that may be made by its manufacturer, is not guaranteed or endorsed by the publisher.
